# Mapping the Global Evidence on Environmental and Climatic Factors Linked to Human Fertility-Related Outcomes: Protocol for a Scoping Review

**DOI:** 10.2196/91589

**Published:** 2026-09-14

**Authors:** Mrunali Zode, Barsha Gadapani Pathak, Reema Mukherjee, Pratibha Dhiman, Amir Sajjad, Vani Kandpal, Richa Kandpal, Priya Karna, Sudipto Roy, Sarmila Mazumder, Tanica Lyngdoh

**Affiliations:** 1 Division of Reproductive, Child Health, and Nutrition, Indian Council of Medical Research, V. Ramalingaswami Bhawan, P.O. Box No. 4911, Ansari Nagar, New Delhi, Delhi, India, 91 11 26588895; 2 Center for Health and Research Development, Society for Applied Studies, New Delhi, Delhi, India; 3 Department of Reproductive, Maternal, Neonatal, Child and Adolescent Health, World Health Organization, New Delhi, Delhi, India; 4 Clinical Studies and Trial Unit, Department of Development Research, Indian Council of Medical Research, New Delhi, India; 5 Faculty of Medical Research, Academy of Scientific and Innovative Research (AcSIR), Ghaziabad, India; 6 Faculty of Biological Sciences, Academy of Scientific and Innovative Research (AcSIR), Ghaziabad, India

**Keywords:** infertility, male infertility, reproductive health, fecundity, environmental exposures, environmental pollution, climate change, endocrine disruptors, Preferred Reporting Items for Systematic Reviews and Meta-Analyses, PRISMA

## Abstract

**Background:**

Increasing attention has been directed toward environmental and climatic exposures as potential factors influencing reproductive function and fertility-related outcomes. However, the available evidence is dispersed across populations, study designs, and exposures.

**Objective:**

The primary objective of this scoping review is to map and synthesize the extent, range, and nature of the global evidence on how environmental and climatic factors influence human fertility outcomes. We aim to capture the environmental and climatic exposures that have been studied, characterize the fertility-related outcomes assessed, and summarize the associations reported between these exposures and outcomes.

**Methods:**

The present scoping review will systematically map global evidence on environmental and climatic factors in relation to human fertility-related outcomes. We will include human studies examining associations between environmental or climatic exposures and fertility-related outcomes, including measures of reproductive function, fertility potential, and reproductive success. A comprehensive search will be conducted across major electronic databases, including PubMed, Embase, Web of Science, and the Cochrane Library. Covidence will be used for screening and data management. Reviewers will independently screen studies for eligibility, with disagreements resolved through discussion or consultation with a third reviewer. Data will be extracted using a standardized form, which will be piloted and refined. Furthermore, the methodological quality of included studies will be appraised using the relevant JBI critical appraisal checklist according to study design. Findings will be synthesized using narrative synthesis, structured tables, and appropriate visual methods. The review will be reported in accordance with the PRISMA-ScR (Preferred Reporting Items for Systematic Reviews and Meta-Analyses extension for Scoping Reviews) guidelines.

**Results:**

Title and abstract screening was completed in May 2026, and full-text screening is underway. Data extraction and synthesis will be conducted after completion of study selection. The review will provide a structured synthesis of available human evidence, summarizing the range of environmental and climatic exposures studied, fertility-related outcomes assessed, and characteristics of included studies. It will also describe the direction and significance of reported findings, including consistent, mixed, or inconclusive evidence, and identify key gaps in the literature to inform future research priorities.

**Conclusions:**

As global fecundity trends evolve and concerns about environmental exposures and climate change intensify, understanding how the environment influences reproductive potential is urgent. This work aligns with global development priorities by addressing the intersection of Sustainable Development Goals 3 (good health and well-being) and 13 (climate action).

## Introduction

### Background

Human fertility is influenced by a complex interplay of biological, behavioral, social, and environmental determinants. In recent years, concerns regarding declining reproductive health indicators, including reduced semen quality in men and evidence of lower ovarian reserve and poorer oocyte quality in women, have contributed to increasing attention to fertility as a global public health issue [1-4]. This is further reflected in the rising demand for assisted reproductive technologies in many settings, suggesting broader challenges with natural conception and reproductive outcomes [5,6].

Fertility and reproductive function are shaped by a wide range of factors, including age, genetics, lifestyle, nutrition, socioeconomic conditions, health care access, and environmental influences. In recent years, environmental and climatic exposures have gained increasing attention in the context of fertility. These include endocrine-disrupting chemicals (EDCs), heavy metals, pesticides, air pollution, and climate-related stressors such as rising temperatures and extreme weather events [7-11]. Such exposures have been associated with hormonal disruption, impaired gamete quality, and altered reproductive function [7,11-15]. In women, environmental exposures have been associated with ovulatory dysfunction, impaired implantation, miscarriage, endometriosis, and earlier menopause [7,12,16,17]. In men, they have been linked to impaired semen parameters, altered reproductive hormone profiles, and increased odds of testicular dysfunction [9,18]. In assisted reproductive settings, higher exposure to environmental pollutants has been associated with lower live birth rates and increased pregnancy loss, with similar patterns also reported among individuals conceiving naturally [19-21].

Although evidence linking environmental and climatic exposures to fertility-related outcomes is growing, it remains dispersed across diverse populations, settings, and study designs. Fertility and reproductive function are multifactorial processes, and fertility-related outcomes vary in their methods of assessment across studies. In addition, environmental and climatic exposures are broad, continuously evolving, and measured using different approaches, limiting comparability across studies. This heterogeneity makes it difficult to synthesize the evidence and obtain a comprehensive understanding of the field.

### Previous Reviews and Rationale for This Scoping Review

Reviews conducted in the past have examined specific environmental exposures in relation to fertility and reproductive health. A systematic review by Checa Vizcaíno et al [22] evaluated the impact of air pollution on human fertility, while another systematic review by Conforti et al [23] focused on air pollution and female infertility. More recently, a systematic review by Segal and Giudice [24] summarized evidence related to reproductive health and environmental stressors, including air pollution, heat stress, floods, wildfires, and toxic chemicals. A scoping review conducted by Heo et al [25] studied the impact of ambient temperature on human infertility. Narrative reviews have also explored environmental toxins in relation to male fertility and female infertility [26,27]. However, the available reviews vary in scope, populations included, and outcomes assessed, with some focusing on specific environmental domains or selected reproductive outcomes, and others including animal, in vitro, and human studies. A broader synthesis is needed to understand the range of environmental and climatic exposures studied in human populations, fertility-related outcomes assessed, study populations and settings included, and areas where evidence remains limited, focusing specifically on evidence from human studies.

As climate change accelerates and environmental degradation continues in many regions, understanding these relationships is increasingly important and timely. Given the broad scope of the topic and the breadth of available evidence, a scoping review approach is appropriate. This manuscript presents a protocol for a scoping review designed to systematically map global evidence on environmental and climatic factors influencing human fertility-related outcomes.

### Objective

The primary objective of this scoping review is to map and synthesize the extent, range, and nature of the global evidence on how environmental and climatic factors influence human fertility outcomes. We aim to capture the environmental and climatic exposures that have been studied, characterize the fertility-related outcomes assessed, and summarize the associations reported between these exposures and outcomes.

## Methods

### Overview

The present protocol was developed in accordance with the Arksey and O’Malley [28] scoping review framework and will be reported following the PRISMA-ScR (Preferred Reporting Items for Systematic Reviews and Meta-Analyses extension for Scoping Reviews) guidelines (Checklist 1) [29]. The review will follow the methodological stages originally outlined by Arksey and O’Malley [28]: (1) identifying the research questions (RQs); (2) identifying relevant studies; (3) selecting eligible studies; (4) charting the data; and (5) collating, summarizing, and reporting the results.

The protocol has been registered with the OSF Registries (osf.io/7tu9s), and any amendments will be documented in the registration record to maintain transparency.

### Stage 1: Identifying the RQs

#### RQs

Guided by the study objective, the review will address the following overarching RQs:

1. Which environmental exposures, their measurement metrics, and exposure windows have been examined in relation to human fertility?
2. What fertility outcomes (eg, clinical pregnancy, time to pregnancy, and live birth) have been examined in relation to those exposures, and how were they defined, measured, or ascertained?
3. What study designs, populations, and settings have been used, and where are the evidence clusters, gaps, and methodological needs (eg, mixtures, personal exposure, and confounding control)?
4. Which environmental exposures and fertility-related outcomes demonstrate consistent associations, and where is the evidence inconclusive?
5. Which populations, geographic regions, exposures, or outcomes are underrepresented in the current evidence base, and what are the implications of these gaps for advancing global equity in fertility research (eg, low- and middle-income countries and vulnerable populations such as occupationally exposed groups)?

The scope of this review is deliberately kept broad to capture all relevant literature, but the questions are clearly defined to maintain focus. We will use the population, concept, and context (PCC) framework (as recommended by the JBI) to clarify the key components of the RQs and eligibility criteria [30]. Textbox 1 summarizes the PCC elements for this review.

Textbox 1. **Population, concept, and context framework for eligibility.**

**Population**

- This will include women of reproductive age (aged 15-49 years), adult men, couples attempting to conceive, individuals in the general population or fertility clinic settings, and early adolescents (aged 10-14 years). Although fertility-related outcomes are commonly assessed during the reproductive years, environmental and climatic exposures that may influence reproductive development and future reproductive function likely begin much earlier in life. Inclusion of the early adolescent age group will allow assessment of environmental exposures examined during early adolescence and their relationship with reproductive function and subsequent fertility-related outcomes. This age represents a critical window of susceptibility during which the reproductive system undergoes rapid maturation. We acknowledge that evidence in this age group may be limited and may include indirect, longitudinal, or retrospective studies.
- There will be no restriction on health status; both individuals who are fertile and individuals who are subfertile or infertile will be included.

**Concept**

- This encompasses any environmental exposure that could plausibly influence human reproductive function. Examples include ambient air pollution, water or soil contaminants, endocrine-disrupting chemicals, industrial and traffic-related emissions, persistent organic pollutants, occupational environmental exposure (eg, farming, mining, and manufacturing), extreme temperatures (eg, heat waves and cold spells), climate factors (eg, humidity, rainfall patterns, and droughts), and broader climate change–related events (eg, heat index trends, wildfires, and flooding) when examined in relation to fertility outcomes.
- We will include any measured fertility-related outcomes, whether at the individual level (eg, clinical pregnancy and time to pregnancy) or population level (eg, birth rate and total fertility rate [TFR]), that reflect reproductive function and fertility potential. These include reproductive outcomes such as pregnancy rate, live birth rate, fecundability, time to pregnancy, infertility prevalence or incidence, and TFR. Measures of reproductive function will also be included, such as semen quality parameters, indicators of ovulatory function, and hormonal markers such as anti-Müllerian hormone, follicle-stimulating hormone, and testosterone. Molecular and cellular markers such as sperm DNA fragmentation and oxidative stress or reactive oxygen species will also be considered. Pregnancy-related outcomes such as miscarriage or recurrent pregnancy loss and stillbirth will be included as important events reflecting failure to achieve or maintain a viable pregnancy.

**Context**

- The review will have a global scope, with no restriction based on geography or setting. We will include evidence from all countries and regions, including low-, middle-, and high-income countries; urban and rural settings; and community-based, occupational, and clinical settings. Studies from all periods up to the present will be included.

#### Exposure and Outcome Definitions

We anticipate a wide range of exposure metrics in the included studies. For this review, environmental and climatic exposures will be defined according to how they were defined and measured in the eligible studies; for example, average ambient temperature, heat index, or extreme heat days; particulate matter with an aerodynamic diameter of ≤2.5 μm (PM_2.5_) concentration in air; and blood levels of heavy metals or endocrine disruptors. We will record the exposure definition, measurement method, exposure period, and relevant thresholds or categories where reported. Fertility-related outcomes will likewise be taken as defined in each study and will be extracted according to the authors’ definitions or criteria, including how infertility was diagnosed or time to pregnancy was measured. By allowing diverse measures, we remain inclusive and align with scoping review best practices that emphasize breadth [31]. However, study-specific exposures and outcomes will be compiled and categorized during data charting based on the characteristics of the available evidence and refined as needed to support meaningful summarization. An indicative list of potential occupational and environmental exposures is presented in Table 1.

This review will focus on both male and female fertility, recognizing that men may be disproportionately affected by environmental exposures due to occupational hazards, industrial pollutants, and lifestyle-related environmental factors. Including male reproductive outcomes allows a comprehensive assessment of how environmental and climatic factors influence fertility across sexes and captures potential differential vulnerability and exposure pathways. An indicative list of potential outcomes is presented in Table 2. This list may be refined following a review of the existing literature.

**Table 1.** List of potential occupational and environmental exposures.

Exposure categories and factors	Description
Ambient air pollution	
Particulate matter (PM_2.5_^a^ and PM_10_^b^)	Mixtures of solid particles and liquid droplets in the air; fine (PM_2.5_) and coarse (PM_10_) particulate matter that may affect respiratory, cardiovascular, and reproductive health [32,33]
Nitrogen dioxide	A gaseous air pollutant primarily generated by traffic emissions and industrial activity and reported in some studies to be associated with reduced fecundability [33]
VOCs^c^	Organic chemicals that are volatile at ambient temperature and can also dissolve in water. They are pervasive in daily life and arise from various sources, including industrial processes, paints, agriculture, and indoor products such as cleaning agents and air fresheners. Exposure to VOCs and their metabolites has been associated with an increased risk of infertility; however, uncertainties remain regarding the strength and consistency of this relationship [34].
PAHs^d^	Organic compounds formed during the incomplete combustion of fossil fuels, coal, wood, oil, gas, solid wastes, petroleum products, and tobacco, as well as through the volatilization of synthetic chemicals, food preparation, and automobile exhaust. PAHs act as EDCs^e^ and have been linked to deleterious changes in the human reproductive system [35]
Soil or water contaminants	
Heavy metals (eg, lead, mercury, cadmium, and arsenic)	Toxic elements found in soil, water, or food sources; associated with endocrine disruption and impaired sperm morphology [36]
Industrial or household contaminants	Includes solvents, EDCs such as perfluoroalkyl and polyfluoroalkyl substances, and other bioaccumulative substances in soil or water with potential reproductive toxicity [37]
Microplastics and nanoplastics	Small synthetic plastic fragments (<0.1 μm for nanoplastics and generally <5 mm for microplastics) arising from degradation of consumer and industrial materials; persistent in soil, aquatic systems, and food chains and linked to impaired semen quality [38,39]
Endocrine-disrupting chemicals	
Pesticides	Agrochemicals, including herbicides, insecticides, and fungicides, that may interfere with hormonal pathways regulating reproduction [40]
Phthalates and bisphenols (eg, bisphenol A)	Synthetic compounds found in plastics, personal care products, and packaging; implicated in altered sperm quality, ovarian function, and hormonal regulation [41]
Parabens, flame retardants, and other consumer-product EDCs	Chemicals used in personal care, furnishings, and electronics; their associations with adverse in vitro fertilization outcomes remain uncertain [42]
Climate and temperature	
Extreme temperatures (eg, heat waves and cold spells)	Acute or chronic exposure to very high or very low ambient temperatures; known to influence sperm quality, menstrual characteristics, and pregnancy outcomes [11,43-45]
Climate factors (eg, humidity, rainfall, droughts, and seasonality)	Meteorological variations that influence human physiology and behavior, potentially affecting conception rates and adverse reproductive events [46]
Broader climate change events	
Heat index trends	A combined measure of air temperature and humidity; prolonged higher values impose heat stress that may impair reproductive function [11,44]
Wildfire smoke	A complex mixture of fine particles, gases, and chemicals from biomass burning that has been associated with declines in sperm quality [47]
Flooding and extreme weather events	Climate-driven disasters that can lead to displacement, contamination of resources, or stress exposures that may disrupt reproductive health [46]

a PM_2.5_: Particulate matter with an aerodynamic diameter of ≤2.5 μm.

b PM_10_: Particulate matter with an aerodynamic diameter of ≤10 μm.

c VOC: volatile organic compound.

d PAH: polycyclic aromatic hydrocarbon.

e EDC: endocrine-disrupting chemical.

**Table 2.** Examples of outcomes and definitions.

Outcomes	Definition
General outcomes	
Primary infertility	Failure to achieve a clinical pregnancy after ≥12 months of regular, unprotected intercourse (≥6 months if woman aged ≥35 years) or clinician-diagnosed infertility
Secondary infertility	Inability to conceive after a previous pregnancy or live birth despite 12 months of unprotected intercourse
Unexplained infertility	Infertility in couples where standard evaluation (ovulation, tubal patency, semen analysis) reveals no abnormality
Subfertility	Reduced probability of conception that does not meet the infertility threshold (eg, prolonged time to pregnancy, recurrent early loss
Outcomes related to female fertility	
Clinical indicators	Measures reflecting the ability to achieve and sustain pregnancy: Fecundability, the probability of achieving pregnancy within one menstrual cycle Time-to-pregnancy, the number of months (or menstrual cycles) from the initiation of unprotected intercourse to the onset of pregnancy Fecundability odds ratio, the ratio of per-cycle conception odds in exposed vs unexposed groups Biochemical pregnancy, positive serum/urine β-hCG without ultrasound confirmation Clinical pregnancy, ultrasound visualization of a gestational sac or definitive evidence of pregnancy Live birth, delivery of a living infant at ≥24 weeks of gestation Early pregnancy loss, pregnancy loss before 12 weeks (includes biochemical loss, miscarriage)
Hormonal alterations	Altered levels of reproductive hormones reflecting ovarian and endocrine function: Anti-Müllerian hormone, a hormone secreted by the granulosa cells of small growing ovarian follicles (especially pre-antral and small antral follicles) and used clinically to assess ovarian reserve in infertility evaluation, predict the response to ovarian stimulation in assisted reproductive technology, support the diagnosis of conditions like polycystic ovary syndrome (where anti-Müllerian hormone may be elevated) Follicle-stimulating hormone Luteinizing hormone Estradiol Progesterone Prolactin
Structural abnormalities	Morphological abnormalities affecting fertility: Antral follicle count, the number of small ovarian follicles, typically measuring 2‐10 mm in diameter, observed and counted via transvaginal ultrasound during the early follicular phase (usually on days 2–5 of the menstrual cycle); these follicles are counted in both ovaries and summed to determine ovarian reserve.
Physiological or functional indicators	Parameters assessing reproductive system performance: Ovulatory function, the ability of the ovary to release a mature oocyte (egg) during each menstrual cycle following normal follicular development and rupture Menstrual cycle regularity, defined as cycles that occur at fairly consistent intervals, usually with a cycle length between 24‐38 days Luteal phase metrics, the period from ovulation to the onset of menstruation, during which progesterone secretion is vital to prepare the endometrium for potential embryo implantation (normal length is 11‐17 days with adequate progesterone secretion) Coital frequency, the number of acts of sexual intercourse within a specified time frame (typically per week or per menstrual cycle) Oocyte yield, the number of mature oocytes retrieved after controlled ovarian stimulation Fertilization rate, the proportion of retrieved oocytes successfully fertilized (pronuclei formation) Embryo quality, morphological grading of the embryo (cell number, fragmentation, blastocyst score) Implantation rate, the number of gestational sacs observed divided by the number of embryos transferred
Outcomes related to male fertility	
Semen parameters	Abnormalities in sperm concentration, total count, motility (total and progressive motility), vitality, morphology, or volume, as per WHO reference standards
Hormonal alterations	Altered levels of reproductive hormones such as testosterone, follicle-stimulating hormone, luteinizing hormone, and inhibin B
Functional or structural abnormalities	Testicular dysfunction, cryptorchidism, varicocele, or altered testicular volume
Molecular and cellular outcomes	Evidence of DNA fragmentation, oxidative stress, or epigenetic changes in sperm

### Stage 2: Identifying Relevant Studies

#### Information Sources

A comprehensive literature search was conducted across PubMed, Embase, Web of Science Core Collection, and the Cochrane Library to identify published studies addressing the review questions. These databases collectively index a wide range of biomedical, public health, environmental science, and cross-disciplinary research. Additionally, we used citation tracking by scanning the reference lists of included articles and pertinent review articles to identify additional studies not captured in the database search.

#### Search Strategy

Relevant vocabulary terms, including MeSH, Emtree terms, and free-text terms identified through preliminary searches, were used to develop the search strategy. The search included phrases related to environmental and climatic exposures, such as “environmental pollution,” “particulate matter,” “heavy metals,” “endocrine-disrupting chemicals,” “climate change,” and “global warming,” combined with fertility outcomes, such as “fertility,” “infertility,” “fecundity,” “reproduction,” “sperm,” “semen,” “pregnancy rate,” “time-to-pregnancy,” and “live birth.” Additional terms related to noise, mobile phone exposure, and processed or preserved foods were considered to capture environmental exposure pathways, including noise pollution, electromagnetic or heat exposure, and preservatives and packaging-related chemicals that may influence fertility-related processes and reproductive function. Search terms were suitably combined using Boolean operators (AND and OR), truncation, and database-specific syntax, and the search strategy was adapted for each database (Multimedia Appendix 1). The review will include human studies published in English from database inception to the date of the search, which was conducted between February 2026 and March 2026, with no geographical restrictions.

The search results from all sources were imported into the systematic review management software Covidence (Veritas Health Innovation) for deduplication and screening. We will update the searches before the final analysis, if necessary, to capture newly published studies up to the point of review completion.

### Stage 3: Study Selection (Screening and Eligibility Criteria)

#### Screening Process

We implemented a 2-level screening process of retrieved records: first by titles and abstracts and then by full text. Reviewers independently screened the title and abstract of each retrieved reference for potential relevance, applying the inclusion and exclusion criteria. At the title and abstract stage, we were overinclusive so that any reference that possibly met the criteria or was unclear was advanced to full-text review. Next, full texts of all potentially relevant studies are obtained and screened independently by a team of reviewers against the eligibility criteria. Appropriate reasons for exclusion at the full-text stage are recorded using a predefined list of categories, such as ineligible population, ineligible study design, ineligible article type, nonhuman study, exposure or outcome of interest not evaluated, study objective not relevant, full text not available, article not available in English, or article could not be traced. This list may be updated iteratively during the review process, if required.

The review team was trained prior to screening and will hold regular meetings to discuss areas of concern, clarify uncertainties, and revisit the eligibility criteria, where required. Any disagreements between reviewers will be resolved through discussion or by consulting a third independent reviewer, as needed. Studies with conflicting decisions between reviewers are flagged as conflicts in Covidence and will be adjudicated by the third reviewer.

We will illustrate the study selection process in a PRISMA (Preferred Reporting Items for Systematic Reviews and Meta-Analyses) flow diagram in the final review report, showing the number of records at each stage of the review process [29].

#### Inclusion and Exclusion Criteria

Studies will be eligible for inclusion if they meet the following criteria: (1) the participants are humans (individuals or couples) in early adolescence or of reproductive age, (2) an environmental or climatic exposure is evaluated or clearly present as a factor, (3) at least 1 fertility-related outcome or indicator of reproductive capability is assessed, and (4) the study design includes observational studies (cohort, case-control, cross-sectional, case series, and ecological studies) or interventional studies if the intervention modifies an environmental exposure.

Animal and in vitro studies, as well as case reports, will be excluded. Studies in which outcomes are limited to maternal or neonatal complications following an established pregnancy that are unrelated to fertility processes or the ability to achieve a live birth (eg, low birth weight, preterm birth, or intrauterine growth restriction) will be excluded. Studies reporting outcomes such as miscarriage or spontaneous abortion, stillbirth, or recurrent pregnancy loss will be included. Studies of environmental or climatic exposures assessed after pregnancy is established will be excluded unless they report outcomes related to the ability to maintain pregnancy or achieve a live birth. Studies focusing on general health outcomes or solely on sexual behavior or contraceptive use rather than fertility will not be included.

Qualitative studies will be excluded unless they report measurable fertility-related outcomes. However, qualitative studies that provide contextual insights into fertility-related processes and environmental exposures may be summarized narratively. Systematic reviews and meta-analyses will not be included as primary sources in data charting to avoid duplication of evidence; however, they will be used for reference mining. Similarly, narrative reviews will also be used to identify additional relevant studies from their reference lists. Editorials, commentaries, and opinion pieces will be excluded unless they report extractable original empirical data relevant to the review objectives.

Eligible studies will be included irrespective of funding source, and information on funding and conflicts of interest will be extracted where available. The review will include studies published in English, with no exclusion based on geographical location, study setting, or publication date.

For potentially relevant studies with only abstract-level information available, or where information is missing or unclear, we will attempt to contact the authors once. If no response is received, these studies will be excluded from data synthesis. For relevant conference abstracts, we will first attempt to identify a corresponding full-text publication. If a full text is unavailable, we will contact the authors once to obtain study details. In the absence of a response, such studies will be excluded from data synthesis.

Given the broad scope, we expect a sizeable number of included studies, and we will synthesize this information by subcategorizing as needed (eg, by exposure type or outcome type during data charting).

### Stage 4: Charting the Data

We will extract and chart data from each included study using a standardized form. A preliminary data charting form is provided as Multimedia Appendix 2, and it will undergo iterative refinement during the review process. We will refer to benchmark articles identified during the full-text review to further refine the data extraction fields, where needed. Independent reviewers will pilot the form on the first 5 to 10 studies and meet to refine the form to ensure that all key data and any emerging relevant variables are captured. The data fields extracted for each study are shown in Table 3.

In addition, information on funding sources and conflicts of interest, as reported, will be extracted (Multimedia Appendix 2).

A team of reviewers will extract data independently from each included study. We will compare the extracted forms for consistency, and any discrepancies will be resolved through discussion and consensus or, where necessary, by consultation with a third reviewer. The finalized data chart for each study will form the basis of our evidence synthesis.

In addition, the methodological quality of included studies will be appraised using the relevant JBI critical appraisal checklist according to study design. Appraisal findings will be descriptively summarized. These findings will help us understand the strengths and limitations of the evidence base and support the interpretation of findings.

**Table 3.** Data fields will be extracted for each study

Data fields	Descriptions
Citation details	We will extract authors, year of publication, journal, and source.
Study characteristics	We will record the country or countries where the study was conducted, setting (eg, community based, fertility clinic, and occupational cohort), study design (eg, cohort, case control, cross-sectional, and randomized controlled trials), sample size, and characteristics of the population (including age, sex, and any specific inclusion criteria such as patients with infertility from clinics or the general population).
Exposures assessed	The environmental or climatic factors of interest, with details on how they were measured. For example, for air pollution exposures, we will note the type of pollutant (eg, PM_2.5_, and nitrogen dioxide) and measurement method (eg, monitoring station data); temperature or climate exposures, we will note the metric (eg, average temperature or number of hot d) and indices such as the heat index or humidity index; and for chemical exposures, we will note the specific chemical or class (eg, pesticide, bisphenol A, and heavy metals) and how exposure was determined (eg, blood level, residential proximity, and job exposure). We will also record exposure levels as reported in the studies (eg, highest vs lowest quartile or data recorded as a continuous variable), whether exposure was measured at the individual or ecological level, and timing (eg, preconception, lifelong, and acute exposure during a specific period).
Fertility outcomes measured	The primary outcomes related to fertility that the study investigated and their definitions. Outcomes will be grouped in a clinically and scientifically relevant manner to enable meaningful synthesis of the available evidence. If multiple outcomes are reported, all relevant outcomes will be extracted.
Key results	We will extract the association measures linking the exposure to the outcome. This will include effect measures (eg, odds ratios, risk ratios, hazard ratios, regression coefficients, and differences in means) with CIs and *P* values for the main exposure-outcome relationships. If a study only reports the qualitative direction (eg, “higher exposure group had lower pregnancy rates”), we will note that.
Secondary findings	If the study examined mechanistic or intermediate end points (eg, hormone changes) in addition to main outcomes, we will capture those as needed to inform interpretation.
Study limitations or indicators of bias	Information on study limitations and any potential indicators of bias reported by authors will be recorded, as these may influence interpretation of findings. This will help inform the discussion of the overall strength of evidence.
Conclusions or author remarks	Conclusions or author remarks will be recorded.

### Stage 5: Collating, Summarizing, and Reporting the Findings

Given the broad scope of this review, evidence synthesis will involve both quantitative mapping and narrative synthesis, in line with scoping review recommendations.

Study characteristics will be summarized in a table detailing study design, geographic location, country income level, study setting, population characteristics, sample size, exposures, and outcomes. The distribution of studies over time and across geographic regions will be illustrated using appropriate figures and maps.

To address RQ 1 and RQ 2, evidence will be mapped to describe the range of environmental exposures examined, their measurement metrics, exposure windows, and fertility-related outcomes assessed, including their definitions and methods used for their ascertainment. Exposure categories and outcome domains will be descriptively categorized. A heatmap matrix will be used to visualize intersections between these exposure categories and outcomes, providing an overview of the most commonly studied exposure-outcome pairs.

Furthermore, to address RQ 4, the consistency of findings for a given exposure-outcome relationship will be assessed descriptively by examining the direction and significance of reported findings across studies. Each exposure-outcome relationship will be summarized using cautious categories such as evidence suggesting an adverse association, no association reported, mixed or inconclusive evidence, limited evidence, and insufficient studies, without formal assessment of effect size or causal inference. These categories will be developed during data charting based on the characteristics of the available evidence and refined as necessary to ensure meaningful synthesis. Findings will be presented using appropriate visual methods. Additionally, evidence will be narratively synthesized by grouping studies by exposure category; describing mechanistic pathways reported in the literature; and highlighting areas of consistent findings, mixed evidence, and evidence gaps.

To address RQ 3 and RQ 5, findings will be mapped by population groups and country income level to identify populations and areas where research has been conducted and where evidence is limited. Geographic regions and country income levels will be classified according to the World Bank classification. Furthermore, to explore whether reported associations differ by study population context, we will stratify findings by setting, distinguishing between clinic-based (hospital cohorts) and community-based (eg, population-based, registry-based, or household cohorts) studies.

Particular attention will be given to study design and methodological characteristics. Occupationally exposed populations will be identified based on study definitions and reported exposure context, where available.

Although the planned approaches to evidence synthesis and presentation are described previously, these may be refined following completion of data extraction based on the nature, breadth, and heterogeneity of the available evidence to ensure that the most appropriate methods are used for summarizing and presenting the findings.

The results will be reported in line with PRISMA-ScR guidelines, including a checklist. In the discussion, emerging patterns in the evidence will be interpreted, along with possible biological mechanisms and public health implications. We will also identify gaps and recommend priorities for future research to guide both science and policy.

### Ethical Considerations

This study is a review of existing published and publicly available data, and we will not access any individually identifiable information. The protocol has been registered on the OSF Registries (osf.io/7tu9s). We will disseminate the findings via a peer-reviewed publication.

## Results

The protocol was submitted to the journal on January 19, 2026. Database searches were completed in the first week of March 2026, followed by completion of title and abstract screening at the end of May 2026. Full-text screening commenced in June 2026, with data extraction and synthesis to be conducted after completion of study selection. The anticipated study timelines are presented in Table 4.

The review is expected to provide a structured synthesis of human evidence on environmental and climatic influences on fertility-related outcomes, summarizing the range of exposures and outcomes studied and key study characteristics such as the populations, settings, study designs, and methods used to define and measure these exposures and outcomes.

The synthesis will also report the direction and significance of findings; identify areas of consistent, mixed, or limited evidence; and highlight policy-relevant gaps in knowledge and priorities for future research.

**Table 4.** Study timeline.

Activities	February 2026	March 2026	April 2026	May 2026	June 2026	July 2026	August 2026	September 2026	October 2026	November 2026
Database search	✓	✓								
Screening		✓	✓	✓	✓	✓				
Data extraction							✓	✓		
Data synthesis and quality assessment									✓	✓
Manuscript preparation										✓

## Discussion

As global fecundity trends evolve and concerns about environmental exposures and climate change intensify, understanding how the environment influences reproductive potential is urgent [48]. This work aligns with global development priorities by addressing the intersection of Sustainable Development Goals 3 (good health and well-being) and 13 (climate action) [49].

This review has some anticipated limitations. Gray literature and non-English publications will not be included, and certain region-specific databases will not be searched, which may prevent the identification of some relevant studies. To mitigate these gaps, the reference lists of included studies and relevant reviews will be screened to maximize evidence capture. We will try to reach out to authors for articles for which the full text is not available. Study quality will be assessed using appropriate, design-specific checklists. Any deviations from the protocol will be documented and reported.

## Supplementary material

10.2196/91589 Multimedia Appendix 1 Search strategies.

10.2196/91589 Multimedia Appendix 2 Preliminary version of the data extraction form.

10.2196/91589 Checklist 1 PRISMA-ScR checklist.

## Abbreviations

EDC: endocrine-disrupting chemical
NO_2_: nitrogen dioxide
PCC: population, concept, and context
PM_2.5_: Particulate matter with an aerodynamic diameter of ≤2.5 μm
PRISMA: Preferred Reporting Items for Systematic Reviews and Meta-Analyses
PRISMA-ScR: Preferred Reporting Items for Systematic Reviews and Meta-Analyses extension for Scoping Reviews
RQ: research question
