# Supplementary material for: Mapping the Global Evidence on Environmental and Climatic Factors Linked to Human Fertility-Related Outcomes: Protocol for a Scoping Review
Source: JMIR Res Protoc. 2026 Sep 14;15:e91589. doi: 10.2196/91589 (PMC13574267; doi:10.2196/91589)
Supplement: Supplementary file 2 [file resprot-v15-e91589-s001.docx]

**Multimedia Appendix 1: Search Strategies**

**1) PubMed**

| **1. Exposure** | "Air Pollution"[MeSH Terms] OR "Environmental Pollution"[MeSH Terms] OR "Climate Change"[MeSH Terms] OR "Global Warming"[MeSH Terms] OR "Heat Waves"[Title/Abstract] OR "Temperature"[MeSH Terms] OR "Ambient Temperature"[Title/Abstract] OR "Extreme Heat"[Title/Abstract] OR "Wildfires"[Title/Abstract] OR "Occupational Exposure"[MeSH Terms] OR "Volatile Organic Compounds"[Title/Abstract] OR "VOCs"[Title/Abstract] OR "Endocrine Disruptors"[MeSH Terms] OR "Phthalic Acids"[MeSH Terms] OR "parabens"[MeSH Terms] OR "bisphenol*"[Title/Abstract] OR "Pesticides"[MeSH Terms] OR "Insecticides"[MeSH Terms] OR "Agrochemicals"[MeSH Terms] OR "Polychlorinated Biphenyls"[MeSH Terms] OR "ddt"[MeSH Terms] OR "pfas*"[Title/Abstract] OR "Flame Retardants"[MeSH Terms] OR "Microplastics"[MeSH Terms] OR "PFOA"[Title/Abstract] OR "Plasticizers"[MeSH Terms] OR "metals, heavy"[MeSH Terms] OR "food, processed"[MeSH Terms] OR "food, preserved"[MeSH Terms] OR "Mobile phones"[Title/Abstract] OR "noise"[MeSH Terms] |
| --- | --- |
| **2. Outcome** | "Fertility"[MeSH Terms] OR "Infertility"[MeSH Terms] OR "infertility, male"[MeSH Terms] OR "infertility, female"[MeSH Terms] OR "Subfertility"[Title/Abstract] OR "Fecundability"[Title/Abstract] OR "Time to Pregnancy"[MeSH Terms] OR "Ovarian Reserve"[MeSH Terms] OR "Semen"[Title/Abstract] OR "Sperm"[Title/Abstract] OR "Semen Quality"[Title/Abstract] OR "Sperm Count"[Title/Abstract] OR "reproductive techniques, assisted"[MeSH Terms] |
|  | #1 OR #2 |
|  | Filters: English, Humans |

**2) Embase**

| **1. Exposure** | exp air pollution/ OR climate change.ti. OR exp heat stress/ OR heat wave*.ti,ab. OR extreme heat.ti,ab. OR noise.ti. OR electromagnetic field*.ti. OR exp endocrine disruptor/ OR exp phthalic acid/ OR exp paraben/ OR bisphenol*.ti,ab. OR pesticide*.ti. OR insecticide*.ti. OR agrochemical*.ti. OR exp polychlorinated biphenyl/ OR exp DDT/ OR PFAS*.ti,ab. OR PFOA.ti,ab. OR plasticizer*.ti. OR flame retardant*.ti. OR  microplastic*.ti,ab. OR volatile organic compound*.ti,ab. OR VOC*.ti,ab. OR heavy metal*.ti. |
| --- | --- |
| **2. Outcome** | exp fertility/ OR exp infertility/ OR exp male infertility/ OR exp female infertility/ OR subfertility.ti,ab. OR fecundability.ti,ab. OR exp time to pregnancy/ OR exp ovarian reserve/ OR semen.ti. OR sperm.ti. OR semen quality.ti,ab. OR sperm count.ti,ab. |
|  | 1 AND 2 |

**3) Web of Science**

| **1. Exposure** | (TS=("air pollution" OR "climate change" OR "global warming" OR "heat stress" OR "heat wave*" OR "extreme heat" OR "endocrine disruptor*" OR "phthalic acid*" OR paraben* OR bisphenol* OR "polychlorinated biphenyl*" OR DDT OR PFAS* OR PFOA OR plasticizer* OR "flame retardant*" OR microplastic* OR "volatile organic compound*" OR VOC*) OR TI=(pesticide* OR insecticide* OR agrochemical* OR "heavy metal*" OR noise OR "electromagnetic field*")) |
| --- | --- |
| **2. Outcome** | (TI=(fertility OR infertility OR "male infertility" OR "female infertility" OR subfertility OR fecundability OR "time to pregnancy" OR "ovarian reserve") OR AB=(fertility OR infertility OR "male infertility" OR "female infertility" OR fecundability OR "time to pregnancy" OR "ovarian reserve") OR TI=(semen OR sperm* OR "semen quality" OR "sperm count" OR "anti-mullerian hormone")) |
|  | 1 AND 2 |

**Cochrane**

| **Exposure AND Outcome** | ("air pollution" OR "climate change" OR "global warming" OR "heat stress" OR  "heat wave*" OR "extreme heat" OR "endocrine disruptor*" OR "phthalic acid*" OR  paraben* OR bisphenol* OR "polychlorinated biphenyl*" OR DDT OR PFAS* OR  PFOA OR plasticizer* OR "flame retardant*" OR microplastic* OR "volatile organic compound*" OR VOC*)  AND  (fertility OR infertility OR "male infertility" OR "female infertility" OR subfertility OR fecundability OR "time to pregnancy" OR "ovarian reserve" OR semen OR sperm* OR "semen quality" OR "sperm count" OR "anti-mullerian hormone") |
| --- | --- |
